# Species Shifts in the *Anopheles gambiae* Complex: Do LLINs Successfully Control *Anopheles arabiensis*?

**DOI:** 10.1371/journal.pone.0031481

**Published:** 2012-03-16

**Authors:** Jovin Kitau, Richard M. Oxborough, Patrick K. Tungu, Johnson Matowo, Robert C. Malima, Stephen M. Magesa, Jane Bruce, Franklin W. Mosha, Mark W. Rowland

**Affiliations:** 1 Kilimanjaro Christian Medical College, Tumaini University, Moshi, Tanzania; 2 National Institute for Medical Research, Amani Medical Research Centre, Muheza, Tanzania; 3 London School of Hygiene and Tropical Medicine, Keppel Street, London, United Kingdom; 4 Pan-African Malaria Vector Research Consortium, Moshi and Muheza-Tanzania, Cotonou-Benin, London School of Hygiene and Tropical Medicine, United Kingdom; 5 RTI International, Centre for Strategic Malaria Solutions, Global Health Group, Nairobi, Kenya; Tulane University, United States of America

## Abstract

**Introduction:**

High coverage of conventional and long-lasting insecticide treated nets (ITNs and LLINs) in parts of E Africa are associated with reductions in local malaria burdens. Shifts in malaria vector species ratio have coincided with the scale-up suggesting that some species are being controlled by ITNs/LLINs better than others.

**Methods:**

Between 2005–2006 six experimental hut trials of ITNs and LLINs were conducted in parallel at two field stations in northeastern Tanzania; the first station was in Lower Moshi Rice Irrigation Zone, an area where *An. arabiensis* predominates, and the second was in coastal Muheza, where *An. gambiae* and *An. funestus* predominate. Five pyrethroids and one carbamate insecticide were evaluated on nets in terms of insecticide-induced mortality, blood-feeding inhibition and exiting rates.

**Results:**

In the experimental hut trials mortality of *An. arabiensis* was consistently lower than that of *An. gambiae* and *An. funestus*. The mortality rates in trials with pyrethroid-treated nets ranged from 25–52% for *An. arabiensis*, 63–88% for *An. gambiae s.s.* and 53–78% for *An. funestus*. All pyrethroid-treated nets provided considerable protection for the occupants, despite being deliberately holed, with blood-feeding inhibition (percentage reduction in biting rates) being consistent between species. Veranda exiting rates did not differ between species. Percentage mortality of mosquitoes tested in cone bioassays on netting was similar for *An. gambiae* and *An. arabiensis*.

**Conclusions:**

LLINs and ITNs treated with pyrethroids were more effective at killing *An. gambiae* and *An. funestus* than *An. arabiensis*. This could be a major contributing factor to the species shifts observed in East Africa following scale up of LLINs. With continued expansion of LLIN coverage in Africa *An. arabiensis* is likely to remain responsible for residual malaria transmission, and species shifts might be reported over larger areas. Supplementary control measures to LLINs may be necessary to control this vector species.

## Introduction

Rapid scaling up of long lasting insecticidal nets (LLINs) has taken place in sub-Saharan Africa, with associated reductions in malaria transmission reported in several countries. The overall effectiveness of LLINs in reducing malaria transmission is indisputable [1], but the relative efficacy of LLINs against different malaria vector species has only partially been explored. Recent data from East Africa showed strong evidence for shifts in sibling species following the scaling-up of ITN/LLINs, with *An. arabiensis* becoming the dominant species in habitats that support sympatric *An. gambiae* and *An. arabiensis* populations. The most notable example was in western Nyanza province, Kenya, where scale-up of ITNs correlated with a proportional decrease in *An. gambiae* from around 85% in 1970–1998 to 1% by 2009 [2]. A similar trend was reported in southern Tanzania where a 79% reduction in *An. gambiae* was documented compared with 38% for *An. arabiensis* following high LLIN coverage [3].

The Pan-African Malaria Vector Research Consortium (PAMVERC) have tested ITNs and LLINs in experimental huts at two locations in NE Tanzania against the three major African malaria vectors: *An. gambiae s.s.*, *An. arabiensis* and *An. funestus* [4], [5], [6], [7], [8]. Efficacy was compared against these species in terms of insecticide-induced mortality, blood-feeding inhibition and induced exiting rates. The findings raise questions about the capacity of LLINs to control *An. arabiensis*.

## Methods

### Rationale

This manuscript compares data from 6 experimental hut trials in which each trial was conducted at two locations in Tanzania, Moshi and Muheza, where *An. arabiensis* and *An. gambiae* occur, respectively. Data on mosquitoes entering the huts at each location was compared in terms of overall mortality, mortality of unfed mosquitoes, blood-feeding (inhibition) rates and exiting rates. The overall mortalities, blood feeding and blood-feeding inhibition rates of free flying mosquitoes resulting from net treatments have previously been individually published [4], [5], [6], [7], [8]. Individual publications are cited in the respective sections. With the re-analysis of the data to extract mortality rates of unfed mosquitoes in comparison with published results the present work illustrates evidence for consistently low mortality of *An. arabiensis* in the presence of ITNs. This may explain the of increasingly noted trends on population shift from *Anoph*e*les gambiae s*.s. to *An. arabiensis* in areas of sympatry.

### Ethics statement

The studies were approved by the Medical Research Coordination Committee of the National Institute for Medical Research, Tanzania, and the London School of Hygiene and Tropical Medicine Ethics Committee (Ref: NIMR/HQ/R.8a/Vol. X/86). Written consent was obtained from all volunteers participating in the study with an understanding of information and identities being kept confidential at all stages of the research and in reporting. During the trial all volunteers were monitored each day for signs of fever or possible side-effects of the ITNs/LLINs.

### Study sites

**Figure 1:**
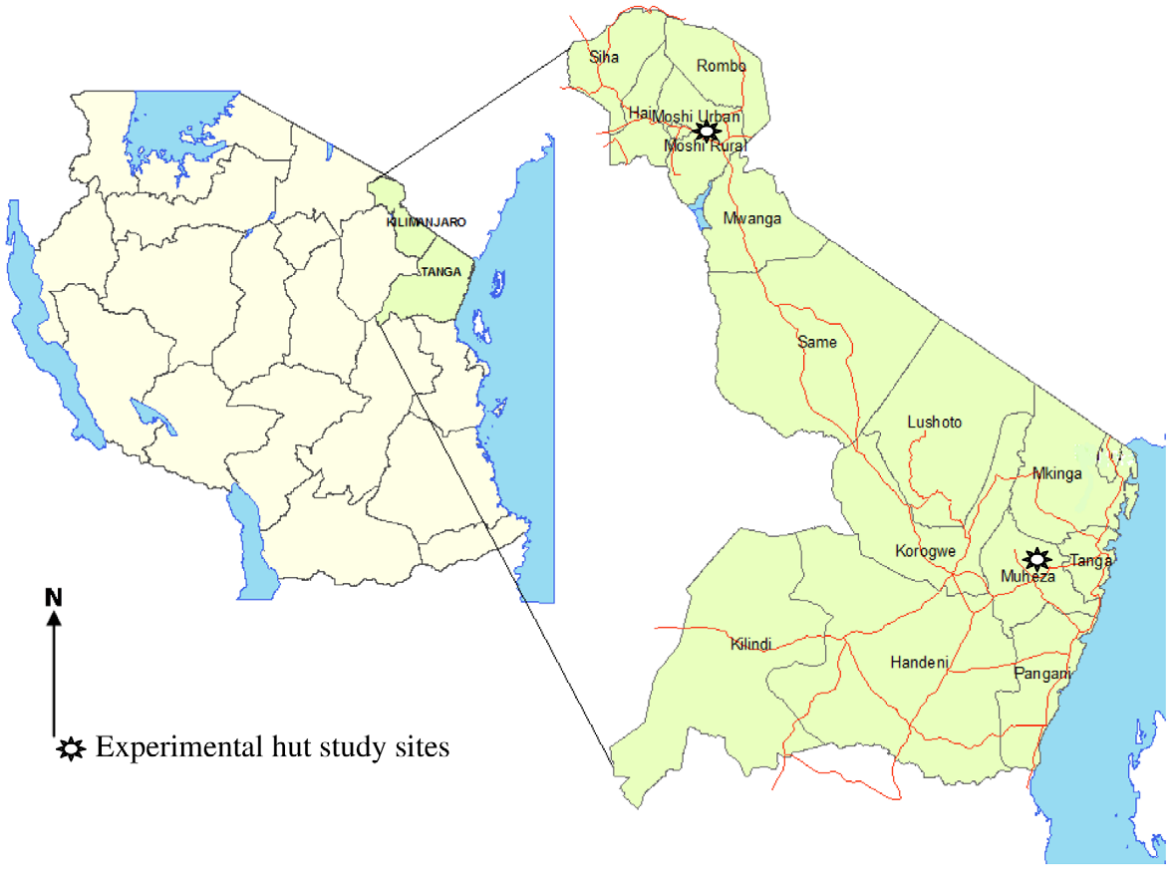
Map showing the location of the field stations, in Moshi and Muheza, north-eastern Tanzania.

This study used suites of experimental huts in two regions of NE Tanzania (Fig. 1). Those of Kilimanjaro Christian Medical College (KCMC) were situated in Lower Moshi Rice Irrigation Zone (3°22′S, 37°19′E; altitude 800 m) which is an irrigated agricultural area surrounded by upland semi-arid steppe. Experimental huts of the Amani Medical Research Centre (NIMR) were situated in Zenet village, Muheza district, (5°13′S, 38°39′E; altitude 193 m) in a lowland coastal region south-west of Tanga City. The two sites differ in terms of malaria transmission and mosquito species composition. The area around Muheza had high levels of *Plasmodium falciparum* malaria transmitted by both *An. gambiae s.s.* and *Anopheles funestus s.s.* Giles mosquitoes. The Moshi site had low levels of *P. falciparum* transmission and *Anopheles arabiensis* Patton is the predominant vector [9]. A modification of polymerase chain reaction method described by Collins *et al* [10] was used to confirm sub-samples of mosquitoes collected from the study sites in Lower Moshi and Zeneth in Muheza. *An. arabiensis* from lower Moshi recorded 80–90% mortality to permethrin (0.75%) test papers, indicating low level pyrethroid resistance [11], and 100% mortality to propoxur (0.1%) test papers indicating susceptibility to carbamates [4]. *An. gambiae* and *An. funestus* from Zeneth recorded 100% mortality to permethrin (0.75%) and propoxur (0.1%) [4].

### Mosquito net treatments

- LLINs & long lasting treatment kits
  1- Olyset; (Sumitomo Chemical Company, Tokyo). Manufactured with 2% permethrin incorporated into polyethylene fibres, corresponding to 1000 mg active ingredient/m^2^.
  2- IconMaxx; (Syngenta Professional Products, Basel, Switzerland) lambdacyhalothrin coated polyester fibre at 50 mg/m^2^.
  3- KO Tab 1-2-3 (Bayer Environmental Sciences, Lyons, France) deltamethrin, coated polyester fibre at 25 mg/m^2^
- ITNs
  4- Deltamethrin (5% SC [suspension concentrate]; Bayer Crop Sciences, Lyons, France) treated polyester fibre at 25 mg/m^2^.
  5- Alphacypermethrin (Fendona SC; BASF Agricultural Products, Limburgerhof, Germany) treated polyester fibre at 25 mg/m^2^.
  6- Carbosulfan (8% SC; FMC Corp., Philadelphia, PA, USA) treated polyester fibre at 200 mg/m^2^.

Conventionally treated nets made of polyester were dipped with target dosages of insecticide according to WHO recommendations [12]. A total of six holes per net measuring 4×4 cm each were cut into all nets to simulate torn nets as is common in local communities [13]. The long lasting insecticide/binder treatments were applied as per manufacturers' instructions.

Target dosages on the ITNs and LLINs was confirmed by the WHO Reference Centre at Gembloux, Belgium, in WHOPES reports of the trials [7], [8] or by high performance liquid chromatography (HPLC) at LSHTM [14].

### Study design

The experimental huts were identical in design at both sites and resembled local housing. They were constructed to a design described by the World Health Organization [13] and others [5], [6], [15] and which was based on the original design of Smith [16] and Smith and Webley [17] for veranda trap huts. There was an eave gap between the wall and roof, a window trap on each wall and a screened veranda on each side.

Adult volunteers slept under nets in each hut from 20:30–6:30. Each morning dead and live mosquitoes were collected from the floors, rooms, verandas and window traps and recorded as blood fed, unfed or gravid. Live mosquitoes were provided with 10% glucose solution under controlled temperature and humidity for 24 h before scoring delayed mortality.

Sleepers were rotated between huts on successive nights to reduce any bias due to differences in individual attractiveness. The treatments were rotated between huts on successive weeks in accordance with a Latin Square design. The direction of two open verandas was routinely changed from East-West to North-South orientation every 2 weeks. Most trials were of 6 week duration with 36 nights of data collection for each type of net.

Species identification done on several samples of *Anopheles gambiae* taken from the sites gave 100% *An. gambiae s.s.* from Zeneth, Muheza (N = 60) and 100% *An arabiensis* from Lower Moshi (N = 60) based on PCR identification results [18]–[20]. All specimens collected in the hut trials identified as members of the *An. gambiae* complex were recorded as *An. arabiensis* in Moshi [18], [19] and as *An. gambiae* in Muheza [20] based on these PCR identification results.

### Data processing and analysis

Data was entered into an Excel database and transferred to Stata 9 for data processing and analysis (Stata Corp LP, College Station, TX, USA). The principal aim of each study was to compare the efficacy of different types of LLIN or ITN (4 pyrethroid and one non-pyrethroid insecticide) as compared to a negative-control untreated net. Data were analysed separately for the two location strata, and therefore different species, Moshi (*An. arabiensis*) and Muheza (*An. arabiensis*, *An. funestus*) as the species were geographically separated. The outcomes of interest were proportion of mosquitoes blood-feeding, dying and exiting on successive nights. Logistic regression for grouped data was used to estimate the outcomes, within each trial, comparing results for treated and untreated nets clustering by day and adjusting for variation between individual sleepers and huts. Estimated proportions were corrected for control mortality using Abbot's correction.

## Results

**Figure 2:**
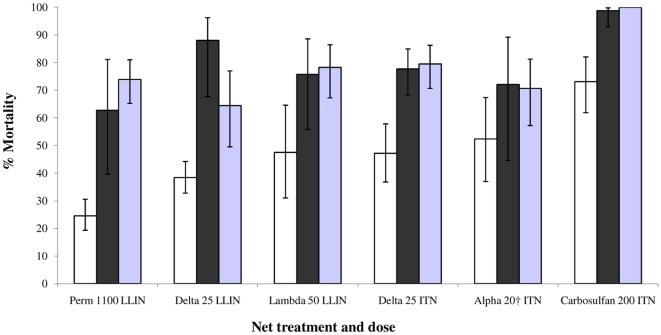
Overall mortality of free flying mosquitoes ±95% confidence interval. *Anopheles arabiensis* (white), *An. gambiae* (black) and *An. funestus* (grey) species.

In all six trials mortality was consistently lower for *An. arabiensis* as compared with the same treatments against *An. gambiae s.s.* and *An. funestus* (Fig. 2). Mortality rates in the untreated control huts was on average 15% for *An arabiensis*, 5% for *An gambiae* and 8% for *An funestus*; in each of the trials the mortality rate in the untreated control was always significantly lower (P<0.001) than in the respective ITN or LLIN treatment. Adjusting the treatment for control mortality made no difference to the trend observed between species; mortality of *An arabiensis* was always lower than that of *An gambiae* (Table 1). The carbamate carbosulfan produced higher mortalities than the pyrethroid treatments but showed similar trends to pyrethroids between species with smaller proportions of *An. arabiensis* being killed. The permethrin (Olyset net) killed the smallest proportions of all. The three alphacyano-pyrethroid treated nets produced rather similar levels of mortality to one another.

**Table 1:** Mortality of mosquitoes freely entering into the huts.

	% mortality of all mosquitoes in treatment arm corrected for control mortality (95% CI)			% mortality of unfed mosquitoes in treatment arm corrected for control mortality (95% CI)		
Type of net	*An. arabiensis*	*An. gambiae*	*An. funestus*	*An. arabiensis*	*An. gambiae*	*An. funestus*
Olyset LN (permethrin)	15 (11–18)	61 (54–69)	72 (66–78)	10 (1–20)	60 (32–82)	74 (63–82)
KO TAB 1-2-3 LN (deltamethrin)	38 (33–43)	88 (75–101)	62 (53–71)	38 (37–41)	90 (66–97)	64 (48–76)
IconMaxx LN (lambdacyhalothrin)	44 (37–51)	74 (59–89)	75 (66–72)	45 (24–65)	72 (49–86)	76 (59–87)
Deltamethrin ITN	29 (23–34)	76 (73–79)	78 (74–83)	32 (15–49)	76 (65–84)	77 (67–85)
Alphacypermethrin ITN	36 (31–40)	71 (62–80)	68 (62–74)	41 (19–60)	71 (39–89)	68 (62–79)
Carbosulfan ITN	67 (63–71)	99 (97–100)	100 (100-100)	65 (46–77)	99 (97–100)	100 (100-100)

Analysis of mortality among unfed mosquitoes collected in the huts gave mortality trends between species similar to that of the combined unfed and blood-fed totals (Table 1). By contrast, cone tests on the treated nets using *An. arabiensis* and *An. gambiae* strains showed equally high mortality rates between *An. arabiensis* and *An. gambiae* (table 2).

**Table 2:** Cone bioassay tests on treated nets using insectary reared mosquitoes.

	% Mortality (no. tested)		
Type of net	*An. arabiensis*	*An. gambiae*	Reference
Olyset LN	83 (55)	99 (100)	[5], [6]
KO TAB 1-2-3 LN	100 (50)	100 (50)	[7]
IconMaxx LN	96 (50)	100 (50)	[8]
Deltamethrin ITN	98 (57)	100 (100)	[4], [6]
Alphacypermethrin ITN	93 (67)	84 (100)	[5], [6]
Carbosulfan ITN	100 (100)	100 (100)	[4]

The proportion of blood-fed mosquitoes was always significantly lower in experimental huts with insecticide-treated nets than with untreated control nets (Table 3). The level of blood-feeding inhibition was similar between all anopheline species. There were no consistent trends between species that suggested insecticide-treated nets were more protective against one species than another.

**Table 3:** Blood feeding rates and insecticide-induced feeding inhibition of free flying Anopheline mosquitoes.

	% Blood-feeding in treatment arm (95% CI)			% Blood feeding inhibition* [% Blood feeding in control arm]			
Type of net	*An. arabiensis*	*An. gambiae*	*An. funestus*	*An. arabiensis*	*An. gambiae*	*An. funestus*	Reference
Olyset LN	22 (15–30)	16 (5–40)	16 (8–29)	60 ^a^ [54]	27 ^c^ [41]	50 ^a^ [32]	[5], [6]
KO TAB 1-2-3 LN	12 (8–16)	20 (6–47)	18 (11–30)	45 ^a^ [74]	64 ^b^ [55]	62 ^a^ [47]	[7]
IconMaxx LN	7 (2–21)	9 (2–34)	20 (12–31)	66 ^a^ [22]	83 ^a^ [53]	47 ^b^ [37]	[8]
Deltamethrin ITN	18 (13–25)	14 (9–20)	10 (5–18)	24 ^d^ [24]	49 ^a^ [26]	79 ^a^ [45]	[4], [6]
Alphacypermethrin ITN	17 (10–27)	9 (4–19)	10 (5–19)	24 ^c^ [31 ^c^]	27 ^b^ [68]	69 ^a^ [32]	[5], [6]
Carbosulfan ITN	25 (21–30)	25 (19–45)	32 (23–43)	39 ^a^ [41]	25 ^d^ [26]	29 ^d^ [26]	[5]

* Superscript letter indicates statistical significance of blood-feeding inhibition in treatment arm as compared to the control arm; a = *P*<0.001, b = *P*<0.01, c = *P*<0.05, d = *P*>0.05.

In the reference huts containing untreated nets the mosquito exiting rates were high for each of the three species (Table 4). Each pyrethroid induced low level exiting of top of this natural inclination to exit by the morning. Far fewer anopheline mosquitoes exited huts with carbosulfan nets, presumably because the majority of mosquitoes were incapacitated by the carbosulfan faster than by pyrethroid.

**Table 4:** Exophily and insecticide-induced exiting of free flying Anopheline mosquitoes.

	% Exophily in treatment arm (95% CI)			% Insecticide-induced exiting* [% Exophily in untreated control arm]		
Type of net	*An. arabiensis*	*An. gambiae*	*An. funestus*	*An. arabiensis*	*An. gambiae*	*An. funestus*
Olyset LN	84 (73–91)	98 (94–99)	93 (85–97)	30 ^a^ [65]	8 ^b^ [91]	2 ^d^ [91]
KO TAB 1-2-3 LN	84 (79–87)	100 (100 - 100)	95 (88–98)	21 ^c^ [91]	10 ^b^ [91]	10 c [87]
IconMaxx LN	87 (77–92)	79 (62–89)	85 (70–93)	6 ^d^ [80]	0 ^d^ [79]	0 ^d^ [89]
Deltamethrin ITN	85 (78–90)	93 (89–96)	94 (88–97)	3 ^d^ [82]	6 ^b^ [88]	16 ^a^ [81]
Alphacypermethrin ITN	84 (80–88)	95 (85–98)	100 (96–100)	2 ^d^ [82]	4 ^d^ [91]	9 ^b^ [91]
Carbosulfan ITN	51 (43–59)	60 (48–71)	45 (35–57)	0 ^a^ [71]	0 ^a^ [88]	0 ^a^ [88]

* Superscript letter indicate statistical significance as compared to the control arm; a = *P*<0.001, b = *P*<0.01, c = *P*<0.05, d = *P*>0.05.

## Discussion

The finding that proportionally fewer *An. arabiensis* than *An. gambiae* were killed by ITNs/LLINs may explain why in areas of sympatric *An. gambiae/arabiensis* populations high coverage has resulted a change in the sibling species ratio (species shifts) in favour of *An. arabiensis* [3], [4].

Species replacement as a result of vector control is not a new phenomenon. Species shifts were recorded in inhabited areas of British Guiana following DDT residual spraying [21]. In this particular example, *Anopheles darlingi*, an anthropophilic major malaria vector was completely eliminated leaving zoophilic *An. aquasalis*, *An. albitarsis*, and *An. triannulatus* unaffected and a persisting residual malaria transmission. A similar shift of species composition was reported during the inception of large-scale IRS in parts of East Africa; this example being the replacement of *An. funestus* Giles with *An. rivulorum* Leeson following IRS with dieldrin in the 1950s [22]. The reasons for this differential effect of IRS on the local vector fauna was attributed to behavioral differences between species which resulted in reduced insecticide contact by *An. rivulorum*. Several hypotheses have been postulated to explain the more recent shifts from *An. gambiae* to *An. arabiensis* including the avoidance of ITNs by *An. arabiensis*, through to preferential feeding on cattle, through to feeding outdoors or feeding earlier [2], [3] whereas, by contrast, *An. gambiae* was considered to be less catholic and more restrictive in its host feeding preferences.

Species identification using PCR confirmed that *An. arabiensis* is to be found in lower Moshi [18], [19] and *An. gambiae s.s* in Muheza [20], [23], [24]. These experimental hut data are the first direct evidence for reduced mortality of host-seeking *An. arabiensis* (compared to *An. gambiae s.s.*) when faced with the barrier of an ITN/LLIN. Conceivably, the lower mortality rates in *An. arabiensis* might be due to an innate process that results in more efficient detoxification of insecticide or, alternatively, to a behavioural trait that results in avoidance of erstwhile lethal dosages. Cone tests on the nets prior to the trials produced rather similar levels of mortality among *An. gambiae* and *An. arabiensis*. Low level permethrin resistance has been recorded in *An. arabiensis* from Lower Moshi [12] but our observervation also extends to alphacyano pyrethroids and to carbosulphan. Differences in intrinsic mortality or capacity to detoxify insecticides between sibling species appear therefore unlikely. Consistent under-dosing of nets in Moshi is another potential, but unlikely explanation for the lower *An. arabiensis* mortality in hut trials, as both trial sites had treated their nets using the same protocols, and the chemical analysis confirmed similar dosages [7], [8], [14], while the Olyset net was factory treated.

Having ruled out intrinsic detoxification and under-dosing, the most likely explanation for lower *An. arabiensis* mortality was behavioral avoidance of treated net surfaces. Notable behavioural differences have been recorded for the two sibling species before. *An. arabiensis* of lower Moshi are highly zoophagic and zoophilic as demonstrated by odour baited traps (OBETs) where odour from cattle attracted 90% of wild free-flying *An. arabiensis* compared to 10% attraction from human odour [25]. *An. gambiae* in Muheza is known to be anthropophilic [5], [6], [23].

An insecticide-treated bed net is essentially a lethal human-baited trap [26]; thus the level of attractiveness to the human is likely to influence mosquito persistence when faced with a physical net barrier and repellency from the insecticide. We hypothesized that zoophilic *An. arabiensis* were less persistent in host seeking when confronted with an ITN and abandoned their attempts to feed earlier than anthropophilic *An. gambiae*, thus a lesser proportion picked up a lethal dose. With that line of argument, if *An. arabiensis* abandoned host-searching more readily than *An. gambiae* when confronted with treated nets one would expect the proportion of blood-feeding inhibition to be higher for *An. arabiensis*. Feeding inhibition turned out to be similar for both sibling species, as was the proportion blood-fed in the untreated control hut. An important factor to consider is the possibility of mosquitoes blood-feeding outdoors either on cattle or humans before entering experimental huts for shelter. If mosquitoes entered huts after feeding outdoors this would bias mortality data as non host-seeking mosquitoes would be less likely to contact treated nets, thus artificially reducing overall mortality. To address this potential confounding factor we analyzed data for unfed mosquitoes. Mortality trends between sibling species for unfed mosquitoes were remarkably similar to trends for total mosquitoes. We also hypothesized that if mosquitoes entered the huts after feeding elsewhere there would be large differences in mortality for fed (low mortality) and un-fed mosquitoes (higher mortality). Mortality data for unfed and blood-fed mosquitoes turned out to be remarkably similar indicating that any mosquitoes entering after feeding from other sources had little impact on overall results.

The low mortality rates observed in *An. arabiensis* cannot be attributed to a greater propensity for exophilic behavior. Contrary to common belief, *An. gambiae* and *An. funestus* showed similar levels of exiting to *An. arabiensis*. ITNs had little effect on proportional exiting rates compared with the control, except for carbosulfan where a large proportion were collected dead in the room having failed to exit before succumbing.

As scale up of LLIN population coverage continues, it is likely that species shifts will be reported over larger areas especially among those *An. arabiensis* populations with higher outdoor feeding tendencies. In some parts of Africa however *An. arabiensis* has been reported to be strongly anthropophagic with proportions fed on humans in indoor resting mosquitoes ranging from 66% to 100% [27], [28], [29], [30]. Such locations are less likely to show species shifts.

Predictive modeling has illustrated the potential impact of climatic change on vector habitat suitability. Increases in maximum and minimum temperature are predicted to have greater impact on habitat suitability for *An. gambiae* than *An. arabiensis*; particularly in Eastern and Southern Africa [31]. However this was ruled out as a potential explanation for the sudden species shift observed in W Kenya which coincided with the rapid ITN/LLIN scale up [2].

Taken together, these factors indicate that in the next decade *An. arabiensis* will be responsible for a greater proportion of malaria transmission in Africa. This does not mean that the vectorial capacity of *An. arabiensis* will increase, merely that the residual malaria transmission may continue through this sibling species while the role of *An. gambiae* sensu strictu diminishes. To further reduce malaria transmission additional tools for the control of *An. arabiensis* may be required; especially in a time where malaria elimination is the target. This could be in the form of *An. arabiensis* specific measures such as insecticide-treated cattle [32] or as an integrated control strategy including larval control [33] or house-screening [34].
